# Patient-Related Risk Factors for Periprosthetic Joint Infection after Total Joint Arthroplasty: A Systematic Review and Meta-Analysis

**DOI:** 10.1371/journal.pone.0150866

**Published:** 2016-03-03

**Authors:** Setor K. Kunutsor, Michael R. Whitehouse, Ashley W. Blom, Andrew D. Beswick, INFORM Team

**Affiliations:** Musculoskeletal Research Unit, School of Clinical Sciences, University of Bristol, Learning & Research Building (Level 1), Southmead Hospital, Southmead Road, Bristol, BS10 5NB, United Kingdom; Rush University Medical Center, UNITED STATES

## Abstract

**Background:**

Periprosthetic joint infections (PJIs) are dreaded complications of total joint arthroplasties. The risk of developing PJIs is likely to be influenced by several patient factors such as sociodemographic characteristics, body mass index (BMI), and medical and surgical histories. However, the nature and magnitude of the long-term longitudinal associations between these patient-related factors and risk of developing PJIs are uncertain.

**Objective:**

To conduct a systematic review and meta-analysis to assess the associations between several patient-related factors and PJI.

**Data Sources:**

MEDLINE, EMBASE, Web of Science, Cochrane Library, and reference lists of relevant studies from inception to September 2015.

**Study Selection:**

Longitudinal studies with at least one-year of follow-up for PJIs after total joint arthroplasty.

**Data Extraction and Synthesis:**

Two investigators extracted data on study characteristics, methods, and outcomes. A consensus was reached with involvement of a third. The relative risk (RR) with 95% confidence intervals was used as the summary measure of association across studies. Study-specific RRs with 95% confidence intervals were meta-analysed using random effect models and were grouped by study-level characteristics.

**Results:**

Sixty-six observational (23 prospective cohort and 43 retrospective cohort or case-control) studies with data on 512,508 participants were included. Comparing males to females and smokers to non-smokers, the pooled RRs for PJI were 1.36 (1.18–1.57) and 1.83 (1.24–2.70) respectively. There was no evidence of any significant associations of PJI with age and high alcohol intake. Comparing BMI ≥ 30 versus < 30 kg/m^2^; ≥ 35 versus < 35 kg/m^2^; and ≥ 40 versus < 40 kg/m^2^; the pooled RRs were 1.60 (1.29–1.99); 1.53 (1.22–1.92); and 3.68 (2.25–6.01) respectively. Histories of diabetes, rheumatoid arthritis, depression, steroid use, and previous joint surgery were also associated with increased risk of PJI. The results remained similar when grouped by relevant study level characteristics.

**Conclusions:**

Several potentially modifiable patient-related factors are associated with the risk of developing PJIs. Identifying patients with these risk factors who are due to have arthroplasty surgery and modulating these risk factors might be essential in reducing the incidence of PJI. Further research is however warranted to assess the potential clinical utility of these risk factors as risk assessment tools for PJI.

**Systematic Review Registration:**

PROSPERO 2015: CRD42015023485

## Introduction

Total joint arthroplasty (TJA) is one of the most common elective orthopaedic surgical procedures; it is a highly successful and cost-effective intervention for alleviating pain and disability associated with advanced joint disease [1–4]. Periprosthetic joint infections (PJIs) are uncommon but devastating and dreaded complications of total joint arthroplasties [5, 6] and can result in severe pain, functional deficits, poor quality of life, and even death [7–9]. PJIs are commonly managed using further surgical revisions (debridements, one- or two-stage revision surgery), which are associated with further risk of re-infection [10], prosthetic complications, subsequent revisions, repeated hospitalizations, and high costs [11, 12]. With increasing life expectancy, a growing healthcare burden due to osteoarthritis [13], and a predicted large rise in the numbers of primary total knee arthroplasties (TKAs) and total hip arthroplasties (THAs) being performed, there may be a proportionate rise in the number of patients requiring revision surgery for PJIs in the coming decades [14, 15].

Given the increasing numbers of patients who will be affected by PJIs and their associated sequelae, which include a high financial burden to health systems [16, 17], it is crucial to address this issue from a public health perspective. Therefore, there is a need to identify risk markers or factors for PJI, which can be modulated to mitigate the risk for developing PJIs in patients. The risk of developing PJI is likely to be influenced by several factors such as characteristics of the patients, the surgical intervention, and post-operative care, but the nature and magnitude of the relationships are uncertain. Several individual studies have reported on the associations of a range of patient-, surgical-, and hospital-related risk factors for PJI, but studies were often poorly powered to adequately quantify the magnitude of the associations. There have been efforts to aggregate these data resulting in a number of published reviews on the topic. In pooled analysis of 14 studies, Kerkhoffs and colleagues reported an odds ratio of 1.90 for overall infection after TKA, comparing obese (body mass index ≥ 30 kg/m^2^) to non-obese patients [18]. Yuan and colleagues also reported a two-fold increase risk of surgical site infections for obesity [19]. Kwong and colleagues found no convincing evidence of a relationship between anticoagulant prophylaxis and PJI in their narrative review [20]. In a meta-analysis of three studies, Tsang and Gaston found diabetes mellitus (DM) to be associated with a 2.04 increased risk of established surgical site infection after elective THA [21]. In the most recent review, Zhu and colleagues in pooled analysis of 14 studies, reported several patient- (e.g. body mass index (BMI), DM, corticosteroid therapy, history of rheumatoid arthritis, malignancy) surgical- (e.g. presence of wound drain, prolonged operative time) and hospital-related factors (e.g. National Nosocomial Infections Surveillance Score ≥ 2; other nosocomial infection) to be associated with risk of PJI [22].

In addition to the limited number of studies pooled which did not provide adequate power to evaluate the associations and the inconclusive results reported by some of these previous reviews, there were several other features of these reviews which limited the validity of their findings. First, most of the reviews (apart from that of Zhu and colleagues [22]) focused on a limited range of potential risk factors, with several of them focusing on obesity [18, 19]. Second, the majority of previous reviews included studies with short follow-up durations (e.g. 30 or 90 days), which precluded the ability to evaluate the long term associations of potential risk factors with the occurrence of PJI. In epidemiological observational studies involving a potential risk factor and an outcome, establishing a long-term association between the risk factor and the outcome enables the development of early intervention strategies in the course of the disease process that may influence the outcome. Third, the heterogeneous definition of infection outcomes by the studies included in these reviews makes interpretation of the findings difficult. Fourth, none of the previous reviews conducted detailed exploration of potential sources of heterogeneity among the contributing studies using formal tests such as subgroup and meta-regression techniques. Fifth, publication bias was not assessed in any of these reviews. Finally, several relevant individual reports have been published since the publication of these reviews. With the emerging evidence on the role of patient-related factors such as sociodemographic characteristics, BMI, and medical and surgical histories in the development of PJI, there is a need to provide robust evidence on the long-term relationships of these patient factors with risk of PJI using large-scale longitudinal data. With the exception of factors such as age and sex, many patient factors are modifiable and could potentially be used in the identification of patients at high risk of developing PJIs and targeting appropriate interventions. In this context, our first aim was to comprehensively and reliably assess the long-term (≥ 1 year follow-up) prospective associations (nature and magnitude) of a wide range of key patient modifiable factors with the risk of PJI using a systematic meta-analytic approach. Our second aim was to address some of the limitations of previous published reviews as reported above. Finally, we also aimed to assess the associations of these patient-related factors with risk of superficial wound infections using a systematic meta-analytic approach.

## Methods

### Data Sources and Search Strategy

This review was conducted using a predefined protocol, which has been registered in the PROSPERO prospective register of systematic reviews (CRD42015023485). It was also conducted in line with PRISMA (Appendix A in S1 File) and MOOSE guidelines [23, 24] (Appendix B in S1 File). We systematically searched MEDLINE, EMBASE, Web of Science, and Cochrane databases from inception up to September 1, 2015. The search strategy combined search terms related to the exposures (e.g., “risk factor”, “body mass index”, “comorbidity”) with those related to the outcomes (e.g., “periprosthetic joint infection”, “prosthetic joint infection”, “deep prosthetic infection”, “deep infection”, “deep surgical site infection”). No restrictions were placed on language, an important consideration, given the perceived international interest in the prevention and management of PJI. The search was complemented by manual scanning of reference lists of identified articles and relevant review articles. The search was restricted to studies conducted in humans. Appendix C in S1 File provides more details on the search strategy.

### Eligibility Criteria

Studies were eligible for inclusion if they were longitudinal studies (prospective or retrospective case-control, prospective cohort, retrospective cohort, case-cohort, nested-case control, or clinical trials) that had reported on the associations of any patient factors such as: (i) sociodemographic characteristics; (ii) BMI; or (iii) medical and surgical histories with PJI following primary or revision TJA (THA, TKA, total shoulder arthroplasty, total elbow arthroplasty, or total ankle arthroplasty), and if they had had at least one year of follow-up (given that a minimum of one-year follow-up after implantation is normally required for PJI surveillance [25–27]). Due to the variation in the definition of surgical site infection in the included studies, outcomes were categorised as PJI (periprosthetic joint infection, deep infection, deep surgical site infection, or deep prosthetic infection) or superficial wound infection. Studies that assessed these patient-related factors and the risk of superficial wound infection with at least one year of follow-up were also included in a subsidiary analysis. We excluded studies (i) that assessed the associations between risk factors and readmissions or revisions due to infection following arthroplasty; (ii) that were restricted to patients with prevalent diseases (e.g. diabetes, rheumatoid arthritis, etc.) and had no comparison or control groups; and (iii) that involved patients who had undergone hemiarthroplasty.

### Data Extraction and Quality Assessment

Two independent authors (S.K.K., A.D.B.) used a standardized data abstraction form to extract data and performed quality assessments. Discrepancies were discussed and agreement reached by adjudication of a third author (M.R.W). Information was extracted on study design, publication year, geographical location, baseline age, proportion of male participants, duration of follow-up, sample size, type of infection, number of infection outcomes, risk estimates (risk ratios or hazard ratios for cohort studies and odds ratios for case-control) for fully-adjusted models when reported, and statistical adjustment for confounders. In the case of multiple publications involving the same cohort, the most up-to-date study or comprehensive study was included. We corresponded with study authors to provide additional information. For studies not published in English, study authors and University colleagues helped translate and interpret the relevant findings. Information on study quality was assessed based on the nine-star Newcastle—Ottawa Scale (NOS) [28], a validated tool for assessing the quality of non-randomised studies (including cohort and case-control studies), which has been described previously [29–31]. Briefly, it incorporates information on three pre-defined domains namely: selection of participants (population representativeness), comparability (adjustment for confounders), and ascertainment of outcomes of interest. The score varies from zero to a maximum of nine, which reflects the highest study quality. A score of ≥ 5 indicated adequate quality for inclusion in our review.

### Statistical Analyses

Relative risks (RRs) with 95% confidence intervals (CIs) were used as summary measures of associations (risk estimates) across studies. Following Cornfield’s rare disease assumption [32], hazard ratios, risk ratios, and odds ratios were assumed to approximate the same measure of RR. Fully-adjusted risk estimates were used when available. Crude RRs were calculated for studies that reported raw counts. To ensure consistency and enhance comparability and interpretation of the findings, reported study-specific RRs (e.g. per 10 unit change) were transformed to a common scale before pooling where possible, using standard statistical methods [33]. Due to the different cut-offs used for BMI by the included studies, we employed the following risk comparisons based on the data available: ≥ 25 versus < 25 kg/m^2^; ≥ 30 versus < 30 kg/m^2^; ≥ 35 versus < 35 kg/m^2^; ≥ 40 versus < 40 kg/m^2^; and per unit increase in BMI. Random-effects models were pre-specified to pool RRs to minimise the effect of between-study heterogeneity [34]. When a study reported more than one estimate of the association between the exposure and outcome (PJI) according to subgroups (e.g., by BMI), a within-study pooled estimate was calculated using a fixed effect analysis. Heterogeneity was quantified using the Cochrane *χ*^*2*^ statistic and the *I*^*2*^ statistic [35]. Subgroup and meta-regression [36] analyses were used to assess several study-level characteristics which might explain heterogeneity, including geographical location, study design, average baseline age, average duration of follow-up, type of arthroplasty, number of infection outcomes, degree of adjustment, and study quality. Evidence of publication bias or small study effects was assessed using funnel plots and Egger’s regression symmetry tests [37]. We performed a narrative synthesis for studies that could not be included in the pooled analysis. All statistical tests were two-sided and p-values of < 0.05 were considered statistically significant. All statistical analyses were performed with STATA release 14 (Stata Corp, College Station, Texas, USA). The dataset for our analyses can be found in S2 File.

## Results

### Study Identification and Selection

**Fig 1:**
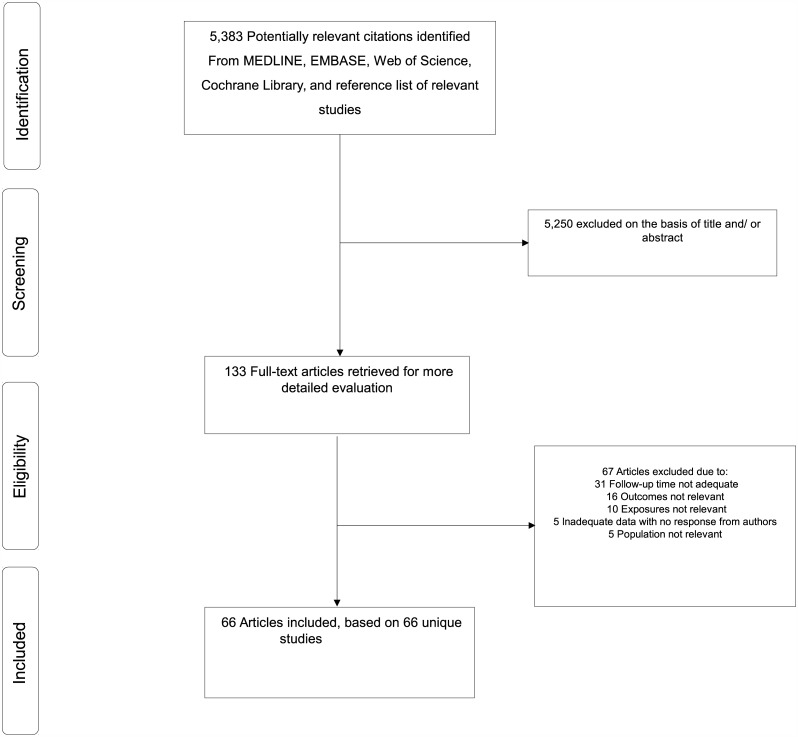
PRISMA flow diagram.

The literature search strategy identified 5,383 potentially relevant articles. After the initial screening of titles and abstracts, 133 articles remained for further evaluation. Following detailed evaluation which included full text reviews, 67 articles were excluded because (i) follow-up time was not adequate (n = 31); (ii) outcomes were not relevant (n = 16); (iii) exposures were not relevant (n = 10); (iv) there was inadequate data with no response from authors (n = 5); and (v) the populations were not relevant (n = 5) (Appendix D in S1 File). The remaining 66 articles corresponding to 66 observational studies met the inclusion criteria and were included in the review (Fig 1; Table A and Appendix E in S1 File).

### Study Characteristics and Study Quality

Table A in S1 File summarizes characteristics and quality assessment scores of the included studies. Overall, the studies involved 512,508 participants, including 8,026 PJI cases. Twenty-six studies each were conducted in Europe (UK, France, Italy, Finland, Spain, Netherlands, Switzerland, Portugal, Belgium, Norway, and Sweden) and North America (USA and Canada); seven in the Pacific (Australia); and seven in Asia (Japan, Korea, China, Hong Kong, Israel, and Malaysia). The mean baseline age of participants ranged from 54 to 72 years. All included studies were observational studies comprising of 23 prospective cohort and 43 retrospective (cohort or case-control) designs with at least one year of follow-up. The mean follow-up for infection outcomes ranged from 1 to 17 years. There was some variability in the type of arthroplasties, but majority of studies involved THAs and TKAs. Methodological quality of included studies ranged from 5–8.

### Associations of Sociodemographic Characteristics and PJI

**Fig 2:**
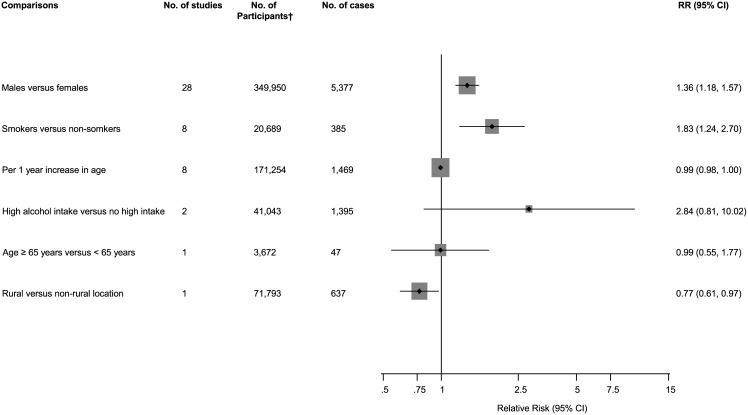
Sociodemographic characteristics comparisons and risk of periprosthetic joint infection. CI, confidence interval (bars); RR, relative risk; †, are number of participants or arthroplasties.

**Fig 3:**
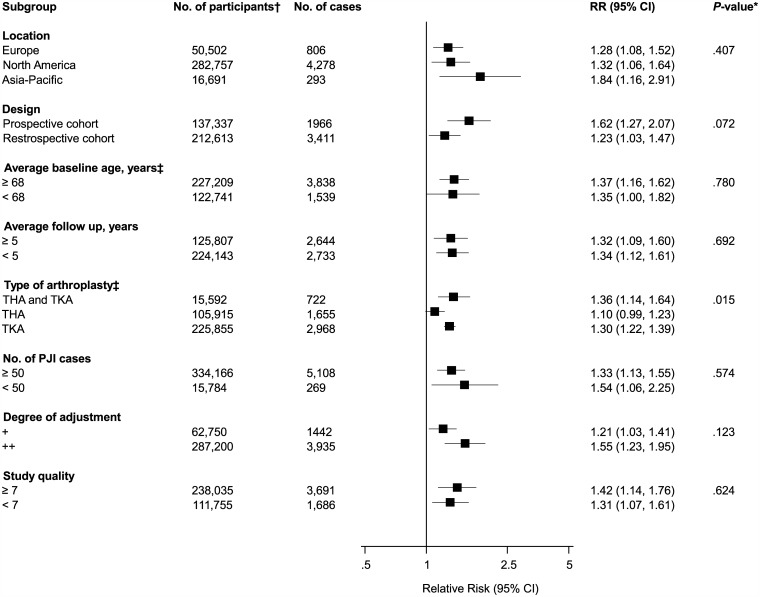
Risk of periprosthetic joint infection comparing males to females, grouped according to several study characteristics. CI, confidence interval (bars); *, *P*-value for meta-regression; †, are number of participants or arthroplasties; ‡, number of cases and participants do not add up to total because of missing data; PJI, periprosthetic joint infection; THA, total hip arthroplasty; TKA, total knee arthroplasty.

The associations of several sociodemographic characteristics with risk of PJI were reported in a total of 32 studies (Fig 2; Figs A-C in S1 File). Comparing males to females in 28 studies, the pooled variably adjusted RR (95% CI) for PJI was 1.36 (1.18–1.57). In pooled analysis of eight studies, smokers had an increased risk of PJI compared to non-smokers RR (95% CI) 1.83 (1.24–2.70). There was no evidence of any statistically significant associations of PJI with age and high alcohol intake. One study reported a decreased risk of PJI comparing patients in rural locations versus non-rural locations, RR (95% CI) 0.77 (0.61–0.97). There was evidence of substantial between-study heterogeneity in the gender analysis (*I*^2^ = 75%; 95% CI 65, 83%; p < 0.001), which was partly explained by type of arthroplasty (*P* for meta-regression = 0.015). Weaker associations were observed in patients with THA compared to studies in patients with TKA (Fig 3).

Two studies (comprising 27,636 participants and 544 cases) reported on the associations of gender with risk of superficial wound infection. Comparing males to females, the pooled RR (95% CI) for superficial wound infection was 0.87 (0.71–1.07).

### Associations of BMI and PJI

**Fig 4:**
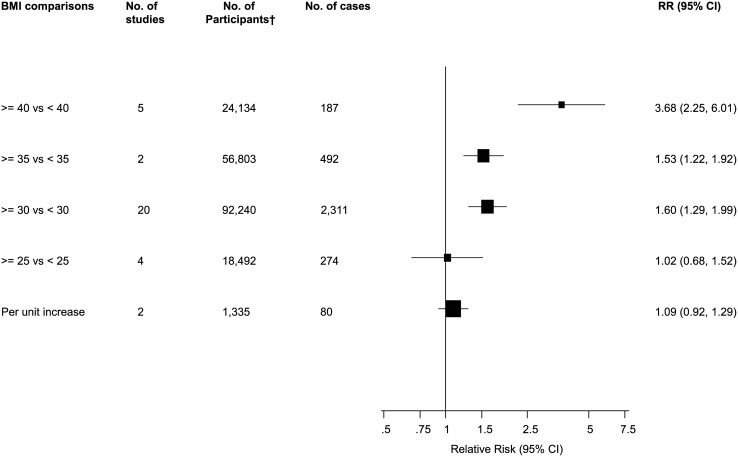
Body mass index comparisons and risk of periprosthetic joint infection. CI, confidence interval (bars); RR, relative risk; †, are number of participants or arthroplasties.

**Fig 5:**
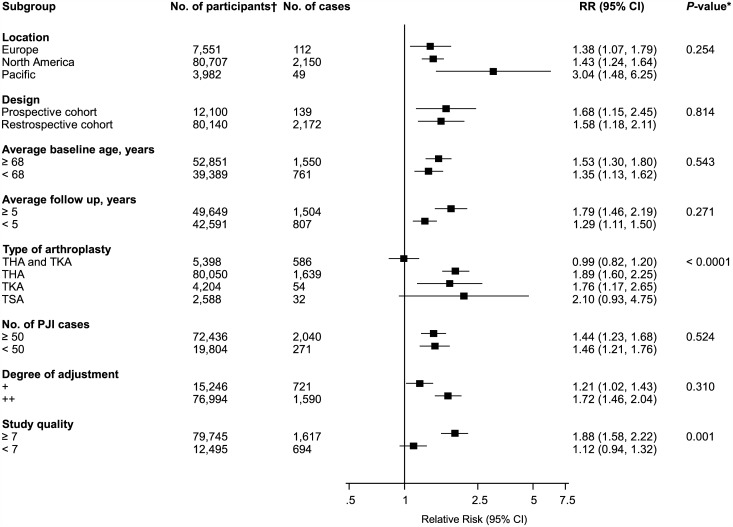
Risk of periprosthetic joint infection in body mass index comparison group ≥ 30 versus < 30 kg/m^2^, grouped according to several study characteristics. CI, confidence interval (bars); *, *P*-value for meta-regression; PJI, periprosthetic joint infection; THA, total hip arthroplasty; TKA, total knee arthroplasty; TSA, total shoulder arthroplasty; †, are number of participants or arthroplasties.

The associations of BMI categories with risk of PJI were reported in 29 studies. The pooled variably adjusted RR (95% CI) for PJI in a comparison of individuals with a BMI ≥ 25 versus < 25 kg/m^2^ was 1.02 (0.68–1.52). Comparing BMIs ≥ 30 versus < 30 kg/m^2^, ≥ 35 versus < 35 kg/m^2^, and ≥ 40 versus < 40 kg/m^2^, the pooled RRs (95% CIs) were 1.60 (1.29–1.99), 1.53 (1.22–1.92), and 3.68 (2.25–6.01) respectively. In pooled analysis of two studies, a unit increase in BMI was associated with a RR (95% CI) of 1.09 (0.92–1.29) for PJI (Fig 4; Fig D in S1 File). There was evidence of moderate between-study heterogeneity in the BMI ≥ 30 versus < 30 kg/m^2^ comparison group (*I*^2^ = 51%; 95% CI 19, 71%; p = 0.004), which was partly explained by study level characteristics such as type of arthroplasty (*P* for meta-regression < 0.0001) and study quality (*P* for meta-regression = 0.001) (Fig 5). Stronger associations were observed in studies that included patients that were treated exclusively by THA or TKA and studies with higher quality scores.

Due to differences in BMI categories, four studies could not be included in the pooled results for PJI. Comparing BMI ≥ 40 versus < 25 kg/m^2^, one study reported a RR (95% CI) of 6.41 (1.67–24.59) for PJI (Reference 55 of Appendix E in S1 File). Another study reported a RR (95% CI) of 18.30 (4.25–79.37) comparing BMI ≥ 50 versus < 50 kg/m^2^ (Reference 37 of Appendix E in S1 File). A further study reported a RR (95% CI) of 3.02 (0.86–10.63) comparing BMI > 35 versus ≤ 35 kg/m^2^ (Reference 11 of Appendix E in S1 File). One study comparing underweight (BMI < 18.5 kg/m^2^) versus normal to overweight BMI category (BMI < 18.5 versus 18.5–30.0 kg/m^2^) reported a RR (95% CI) of 2.88 (0.89–9.23) (Reference 52 of Appendix E in S1 File).

Ten studies reported the associations of BMI categories with risk of superficial wound infection. In pooled analysis of six studies, the RR (95% CI) for superficial wound infection in a comparison of individuals with a BMI ≥ 30 versus < 30 kg/m^2^ was 2.78 (1.77–4.35). In pooled analysis of three studies comparing BMIs ≥ 40 versus < 40 kg/m^2^, the RR was 2.81 (1.44–5.50) in pooled analysis of three studies (Fig E in S1 File). There was no evidence of heterogeneity between contributing studies in both analyses. One study could not be included in the pooled analysis. In this study, a RR (95% CI) of 9.89 (1.03–94.82) for superficial wound infection comparing BMI > 35 versus ≤ 35 kg/m^2^ was reported (Reference 11 of Appendix E in S1 File).

### Medical and Surgical Histories and Risk of PJI

**Fig 6:**
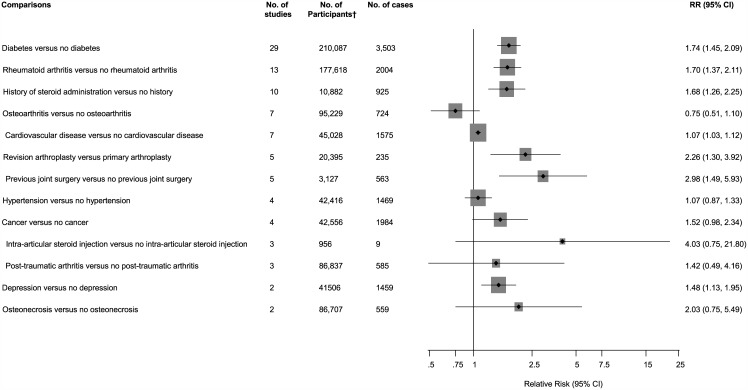
Medical and surgical history comparisons and risk of periprosthetic joint infection. CI, confidence interval (bars); RR, relative risk; †, are number of participants or arthroplasties.

Forty-two studies reported on the associations between several medical and surgical history characteristics with risk of PJI. Comparing patients with histories of diabetes versus no diabetes, rheumatoid arthritis versus no rheumatoid arthritis, and depression versus no depression, the pooled variably adjusted RRs (95% CIs) for PJI were 1.74 (1.45–2.09), 1.70 (1.37–2.11), and 1.48 (1.13–1.95) respectively. Patients with a history of steroid administration versus no history of steroid administration and previous joint surgery versus no previous joint surgery had an increased risk of PJI, RRs (95% CIs) 1.68 (1.26–2.25) and 2.98 (1.49–5.93) respectively. Patients who had a revision arthroplasty had an increased risk of PJI compared to those who had a primary arthroplasty, RR (95% CI) 2.26 (1.30–3.92). There was no evidence of any statistically significant associations of PJI with histories of osteoarthritis, osteonecrosis, post-traumatic arthritis, cardiovascular disease (CVD), hypertension, and cancer. Use of intra-articular steroid injection was also not statistically significantly associated with an increased risk of PJI (Fig 6; Figs F-K in S1 File). Two studies could not be included in the pooled analysis. In one study, there was an increased risk of PJI comparing frail patients to non-frail patients, RR (95% CI) 1.64 (1.28–2.11) (Reference 62 of Appendix E in S1 File). In another, the authors reported no evidence of any statistically significant associations of PJI with low- or high-risk dental procedures (Reference 39 of Appendix E in S1 File). There was evidence of substantial between-study heterogeneity in the diabetes analysis (*I*^2^ = 72%; 95% CI 59, 81%; p < 0.001), which was partly explained by event rate for PJI (*P* for meta-regression = 0.015). Stronger associations were observed in studies with < 50 cases compared to studies with ≥ 50 cases (Fig L in S1 File).

Seven studies reported on the associations of diabetes status, previous joint surgery, and use of intra-articular steroid injection with risk of superficial wound infection. In patients with diabetes compared with those with no diabetes, the RR (95% CI) for superficial wound infection in pooled analysis of three studies (2,020 participants and 61 cases) was 2.57 (1.07–6.17). There was no statistically significant evidence of any associations with previous joint surgery and use of intra-articular steroid injection. There was no evidence of heterogeneity between contributing studies in these analyses.

### Publication Bias

Funnel plots for all analyses that involved five or more studies were all symmetrical under visual examination, with the exception of studies that evaluated associations of PJI with diabetes status. The results were consistent with Egger’s regression tests showing little evidence of publication bias for all analyses except for diabetes status and PJI (Fig M in S1 File).

## Discussion

### Key Findings

In a single comprehensive investigation, we have systematically reviewed and summarised through a meta-analytical approach, available observational longitudinal studies that have assessed associations of several patient-related (sociodemographic factors, BMI, and medical and surgical histories) characteristics with risk of PJI. For sociodemographic characteristics, our results showed an increased risk of PJI in males compared to females and in smokers compared to non-smokers. There was however no statistically significant association of age or high alcohol intake with risk of PJI. One study reported a decreased risk of PJI comparing patients in rural locations to those in non-rural locations. For BMI, consistently statistically significant positive associations were demonstrated for BMI comparisons that involved cut-offs of > 30 kg/m^2^ or higher. One study that compared underweight versus normal to overweight BMI category, reported a trend towards increased risk of PJI for the underweight category, but the estimate was imprecise and not statistically significant. In the analyses of medical and surgical histories; diabetes, rheumatoid arthritis, depression, history of steroid administration, and previous joint surgery were each found to be associated with an increased risk of PJI after THA or TKA. One study reported an increased risk of PJI after TKA in frail compared with non-frail patients.[38] There was no evidence of any statistically significant associations of PJI with histories of osteoarthritis, osteonecrosis, post-traumatic arthritis, CVD, hypertension, cancer, and use of intra-articular steroid injection.

In the subsidiary analyses of patient-related risk factors and superficial wound infection, only BMI (≥ 30 kg/m^2^) and history of diabetes were found to be associated with the risk of superficial wound infection.

### Comparison with Previous Work

Some of our findings concur generally with that of previous reviews on the topic. We also provide several relevant findings that have not been previously addressed. Our findings of an increased risk of PJI comparing obese to non-obese patients concurs with previous work on the topic [18, 19]. However, we have also demonstrated this using several BMI categories and also reported on the associations with superficial wound infection. In contrast to the reviews by Tsang and Gaston [21] and Zhu and colleagues [22] which reported DM to be associated with an increased risk of PJI in pooled analysis of up to eight studies, we demonstrated this association in 29 studies and robustly showed that the risk of PJI remained broadly similar across several clinically relevant subgroups. Chen and colleagues in a meta-analysis of 12 cohort or case-control studies involving 548 PJIs, reported several patient-related factors such as male gender, BMI > 30 kg/m^2^, diabetes, osteoarthritis, hypertension, steroid therapy, and RA to be associated with deep infection [39]. We found no association of PJI with osteoarthritis and hypertension. Similar to our findings, they also showed that age was not associated with the risk of PJI. In the recent elegant review by Zhu and colleagues [22], in addition to a number of patient-related factors (such as hypoalbuminaemia, immunodepression, and coagulopathy) which were observed to be associated with PJI, the authors also evaluated the associations of several surgical- and hospital-related factors with the risk of PJI. In agreement with their findings, BMI, DM, steroid therapy, history of rheumatoid arthritis, and previous surgery were found to be associated with risk of PJI; whiles no associations were demonstrated for high alcohol intake, hypertension, and CVD. However, despite the wide-ranging array of potential risk factors evaluated, the meta-analysis was based on only 14 studies retrieved from multiple databases spanning 1980–2014, with majority of the associations based on pooled analysis of 2–3 studies. In addition, in contrast to the approach employed by these previous reviews, we assessed the long-term associations (follow-up duration of ≥ 1 year) between these factors and the risk of PJI. We also conducted subgroup analyses by relevant study level characteristics. In our analyses comparing males to females, the risk of PJI remained broadly similar across several clinically relevant subgroups. However, stronger associations were observed in studies that included patients with a combination of THA and TKA compared to patients treated exclusively by THA or TKA. Comparing BMI ≥ 30 versus < 30 kg/m^2^ (which had the highest number of studies in this comparison), the risk of PJI remained broadly similar across several clinically relevant subgroups.

### Possible Explanations for Findings

A number of postulated mechanisms have been postulated for some of the associations demonstrated. Patient-related factors that are known to be causally related to PJI include male sex and previous joint surgery [40]. Smoking has been suggested to contribute to PJI by delaying wound healing through nicotine-mediated vasoconstriction [41, 42]. A sluggish circulation causes tissue hypoxia with increased susceptibility to infection [40]. In addition, smoking causes endothelial dysfunction, inflammation, progression of atherothrombosis, and impaired systemic immune response, which are known to contribute to poor wound healing and subsequently to infection. High alcohol intake, similar to smoking, is known to lead to higher postoperative complications including PJI. We were unable to demonstrate this in our study, possibly due to the low power to detect an effect in our analysis (two studies), evidenced by our imprecise estimate. Obesity (BMI > 30 kg/m^2^) has consistently been recognized as an independent risk factor for PJI. Suggested underlying factors for this increased risk include long operative times and presence of additional comorbidities [43, 44]. Obese people are also known to have impaired tissue antibiotic penetration [45]. One study included in our review suggested an association between low BMI and increased risk of PJI, which may be due to underlying poor nutritional status and impaired immunity [46]. The association between diabetes and PJI may be mediated by impaired leukocyte function and microvascular complications [47], which may impair wound healing. In addition, factors associated with diabetes, such as hyperglycemia, hyperlipidemia, hypertension, and increased oxidative stress, upregulate cellular and inflammatory reactions and play a part in atherothrombosis [48], which also cause impaired wound healing. Hyperglycemia, has also been shown to increase biofilm formation [49], which may cause an increased risk of PJI. Given that there is a genetic predisposition to PJI [50], whether some of these associations have any causal relevance may need to be confirmed in appropriate interventional studies or Mendelian randomization studies [51].

### Implications of Our Findings

Our results are very relevant and offer new insight concerning the relationships between several patient-related risk factors and PJI risk. The findings may also have implications for the prevention of PJI after total joint arthroplasty. Given the challenging complication of PJI and its major burden for patients [9] and health systems [52], prevention through implementation of effective strategies is the first and best strategy and should be a priority. Therefore, identifying, mitigating, and optimising amenable risk factors for PJI is a highly desirable approach for prevention. Our findings show that multiple potential modifiable patient-related factors such as smoking, BMI ≥ 30 kg/m^2^, diabetes, depression, steroid use, and frailty are significantly associated with long-term risk of PJI. Identifying patients with these risk factors who are due to have arthroplasty surgery and providing interventions to modify these risk factors might form the basis of PJI prevention strategies. For example, it has been established that smoking cessation before surgery is associated with more than 50% decreased risk of postoperative infections [53]. Indeed, some centers in line with US Centers for Disease Control and Prevention (CDC) recommendations [26], enrol smokers in a cessation program and instruct them to abstain from smoking for at least 30 days before elective arthroplasty [54]. Randomized controlled trials are also warranted to investigate the potential implications.

### Study Strengths and Limitations

The current study has several advantages compared to previous reviews. Given the inclusion of several recent published reports on the topic, it is an updated assessment of the topic. Our search strategy was comprehensive, was without language restriction, and spanned multiple databases, yielding 66 published reports on the topic, making it the largest review on the topic to date. Compared to previous reviews, we had enhanced power to examine the associations of multiple patient-related factors with risk of PJI, as well as superficial wound infection in greater detail. We only included studies with at least one year follow-up for infection outcomes, reliably indicating long-term associations between these factors and risk of PJI. Our results were robust as we were able to standardize and harmonize reported associations (to a common scale) from almost all contributing studies before pooling, thereby quantifying more reliably the magnitude of the associations between several risk factors and PJI. We also conducted detailed subgroup analyses using a range of several study-level and clinically relevant characteristics to explore potential sources of heterogeneity. Formal tests including subgroup analysis by study size, were unable to detect publication bias for a majority of the analyses. The limitations of the present review also merit careful consideration. First, due to lack of a standard definition of PJI in included studies, it was not possible to attain a consistent definition across all studies. We however conducted a detailed assessment using a validated tool for assessing the quality of non-randomised studies, to provide an unbiased assessment of PJI outcomes. Second, we pooled estimates from both retrospective and prospective cohort designs, which could have potentially led to biased estimates. However, subgroup analyses according to the type of study design showed generally similar associations. Third, we were unable to test for the dose-response shapes of the associations between patient-related risk factors (such as BMI) and PJI. Fourth, there was evidence of substantial heterogeneity among contributing studies for some analyses, however we systematically investigated for possible sources of heterogeneity using several subgroup and meta-regression analyses. Fifth, as the present review was based on variably adjusted data reported by the eligible studies, there remained a risk of residual confounding. However, subgroup group analyses in relevant comparisons showed similar risks of PJI between unadjusted and multivariate-adjusted data. Sixth, majority of studies reported on PJI following TJA and did not make a distinction between THA and TKA, however, our subgroup analyses explored the associations for this distinct groups where adequate data allowed. Seventh, majority of studies also failed to make a distinction between primary and revision TJA, however, we were able to assess the associations comparing patients who had a revision arthroplasty versus those who had a primary arthroplasty. Eight, it was not clear from majority of included studies that “history of steroid administration” included a combination of oral steroid administration and use of intra-articular steroid injection. However, we were able to pool estimates from three studies based on use of intra-articular steroid injection. Finally, we were unable to evaluate the associations of diabetes-related (e.g. duration and severity of diabetes) and smoking-related (e.g. frequency of smoking) factors on the risk of PJI given the sparse data available. In one of the included studies, Moon and colleagues demonstrated that the duration of diabetes and the number of complications of diabetes did not influence the risk of PJI [55]. Further studies are warranted to evaluate the associations of these related factors with risk of PJI. Given the several limitations of aggregate data, access to individual participant data should enable a consistent definition of PJI outcomes and a detailed assessment of several aspects of the associations such as characterisation of the shape of any dose-response relationships, consistent adjustment for covariates across studies, assessment of the associations in different subgroups, and the usefulness of patient-related risk factors in PJI risk prediction. The findings should therefore be interpreted in context of the limitations described.

## Conclusions

Evidence from aggregate published data suggests that several potential amenable patient-related factors such as smoking, BMI ≥ 30 kg/m^2^; diabetes, depression, steroid use, and frailty are associated with long-term risk of developing PJIs. Identifying patients with these risk factors who are due to have arthroplasty surgery and modulating these risk factors might be essential in reducing the incidence of PJI. Further research is however warranted to assess the potential clinical utility of these risk factors as risk assessment tools for PJI.

## Supporting Information

S1 File (DOC)

S2 File (CSV)
