# Supplementary material for: Patient-Related Risk Factors for Periprosthetic Joint Infection after Total Joint Arthroplasty: A Systematic Review and Meta-Analysis
Source: PLoS One. 2016 Mar 3;11(3):e0150866. doi: 10.1371/journal.pone.0150866 (PMC4777569; doi:10.1371/journal.pone.0150866)
Supplement: S1 File — (DOC) [file pone.0150866.s001.doc]

**S1 File**

| **Appendix A** | PRISMA checklist |
| --- | --- |
| **Appendix B** | MOOSE checklist |
| **Appendix C** | Literature search strategy |
| **Appendix D** | Reference list of excluded studies |
| **Appendix E** | Reference list of studies included in review |
| **Table A** | Characteristics of studies included in review |
| **Figure A** | Relative risks of periprosthetic joint infection comparing males to females |
| **Figure B** | Relative risks of periprosthetic joint infection comparing smokers to non-smokers |
| **Figure C** | Relative risks of periprosthetic joint infection per 1 year increase in age |
| **Figure D** | Relative risks of periprosthetic joint infection by body mass index comparisons |
| **Figure E** | Relative risks of superficial wound infection by body mass index comparisons |
| **Figure F** | Relative risks of periprosthetic joint infection comparing patients with diabetes versus no diabetes |
| **Figure G** | Relative risks of periprosthetic joint infection comparing patients with rheumatoid arthritis versus no rheumatoid arthritis |
| **Figure H** | Relative risks of periprosthetic joint infection comparing patients with steroid use versus no steroid use |
| **Figure I** | Relative risks of periprosthetic joint infection comparing patients with osteoarthritis versus no osteoarthritis |
| **Figure J** | Relative risks of periprosthetic joint infection comparing patients with cardiovascular disease versus no cardiovascular disease |
| **Figure K** | Relative risks of periprosthetic joint infection for other medical and surgical history characteristics |
| **Figure L** | Risk of periprosthetic joint infection patients with diabetes versus no diabetes, grouped according to several study characteristics |
| **Figure M** | Assessment of small study effects by funnel plots and Egger’s regression symmetry tests |

**Appendix A.** PRISMA checklist

| **Section/topic** | **Item No** | **Checklist item** | **Reported on page No** |
| --- | --- | --- | --- |
| **Title** | | | |
| Title | 1 | Identify the report as a systematic review, meta-analysis, or both | 1 |
| **Abstract** | | | |
| Structured summary | 2 | Provide a structured summary including, as applicable, background, objectives, data sources, study eligibility criteria, participants, interventions, study appraisal and synthesis methods, results, limitations, conclusions and implications of key findings, systematic review registration number | 2 |
| **Introduction** | | | |
| Rationale | 3 | Describe the rationale for the review in the context of what is already known | 4-5 |
| Objectives | 4 | Provide an explicit statement of questions being addressed with reference to participants, interventions, comparisons, outcomes, and study design (PICOS) | 5 |
| **Methods** | | | |
| Protocol and registration | 5 | Indicate if a review protocol exists, if and where it can be accessed (such as web address), and, if available, provide registration information including registration number | 2 |
| Eligibility criteria | 6 | Specify study characteristics (such as PICOS, length of follow-up) and report characteristics (such as years considered, language, publication status) used as criteria for eligibility, giving rationale | 6 |
| Information sources | 7 | Describe all information sources (such as databases with dates of coverage, contact with study authors to identify additional studies) in the search and date last searched | 6 |
| Search | 8 | Present full electronic search strategy for at least one database, including any limits used, such that it could be repeated | Appendix Supplement C |
| Study selection | 9 | State the process for selecting studies (that is, screening, eligibility, included in systematic review, and, if applicable, included in the meta-analysis) | 6-7 |
| Data collection process | 10 | Describe method of data extraction from reports (such as piloted forms, independently, in duplicate) and any processes for obtaining and confirming data from investigators | 6-7 |
| Data items | 11 | List and define all variables for which data were sought (such as PICOS, funding sources) and any assumptions and simplifications made | 6-7 |
| Risk of bias in individual studies | 12 | Describe methods used for assessing risk of bias of individual studies (including specification of whether this was done at the study or outcome level), and how this information is to be used in any data synthesis | 7-8 |
| Summary measures | 13 | State the principal summary measures (such as risk ratio, difference in means). | 7-8 |
| Synthesis of results | 14 | Describe the methods of handling data and combining results of studies, if done, including measures of consistency (such as I^2^ statistic) for each meta-analysis | 7-8 |
| Risk of bias across studies | 15 | Specify any assessment of risk of bias that may affect the cumulative evidence (such as publication bias, selective reporting within studies) | 7-8 |
| Additional analyses | 16 | Describe methods of additional analyses (such as sensitivity or subgroup analyses, meta-regression), if done, indicating which were pre-specified | 7-8 |
| **Results** | | | |
| Study selection | 17 | Give numbers of studies screened, assessed for eligibility, and included in the review, with reasons for exclusions at each stage, ideally with a flow diagram | 8 and Fig. 1 |
| Study characteristics | 18 | For each study, present characteristics for which data were extracted (such as study size, PICOS, follow-up period) and provide the citations | 8-9, Table A |
| Risk of bias within studies | 19 | Present data on risk of bias of each study and, if available, any outcome-level assessment (see item 12). | 9-10, Table A |
| Results of individual studies | 20 | For all outcomes considered (benefits or harms), present for each study (a) simple summary data for each intervention group and (b) effect estimates and confidence intervals, ideally with a forest plot | 9-10, Figs. 2, 4, and 6; |
| Synthesis of results | 21 | Present results of each meta-analysis done, including confidence intervals and measures of consistency | 9-10, Figs. 2, 4, and 6; Figures A-K |
| Risk of bias across studies | 22 | Present results of any assessment of risk of bias across studies (see item 15) | 9-10 |
| Additional analysis | 23 | Give results of additional analyses, if done (such as sensitivity or subgroup analyses, meta-regression) (see item 16) | 9-10; Figs. 3 and 5; Figures L and M |
| **Discussion** | | | |
| Summary of evidence | 24 | Summarise the main findings including the strength of evidence for each main outcome; consider their relevance to key groups (such as health care providers, users, and policy makers) | 10-11 |
| Limitations | 25 | Discuss limitations at study and outcome level (such as risk of bias), and at review level (such as incomplete retrieval of identified research, reporting bias) | 11-12 |
| Conclusions | 26 | Provide a general interpretation of the results in the context of other evidence, and implications for future research | 12 |
| **Funding** | | | |
| Funding | 27 | Describe sources of funding for the systematic review and other support (such as supply of data) and role of funders for the systematic review | 13 |

**Appendix B.** MOOSE checklist

**Patient-related risk factors for periprosthetic joint infection after total joint arthroplasty: A systematic review and meta-analysis**

| **Criteria** | | **Brief description of how the criteria were handled in the review** |
| --- | --- | --- |
| **Reporting of background** | |  |
| √ | Problem definition | The risk of developing periprosthetic joint infections (PJIs) after total joint arthroplasties are likely to be influenced by several patient factors such as sociodemographic characteristics, and previous medical and surgical histories. However, the nature and magnitude of the long-term prospective associations between these patient factors and risk of developing PJIs are uncertain. In this context, we have carried out a systematic review and meta-analysis of longitudinal studies to comprehensively assess the associations between several patient-related risk factors and PJI |
| √ | Hypothesis statement | Several patient-related factors are associated with PJIs after total joint arthroplasty. |
| √ | Description of study outcomes | Periprosthetic joint infection (surgical site infection, superficial wound infection, or deep prosthetic infection) |
| √ | Type of exposure | (i) Sociodemographic factors – age, sex, body mass index, socioeconomic status, smoking status, tobacco use, alcohol consumption  (ii) Previous medical and surgical history – history of diabetes, history of hypertension, comorbidities, rheumatoid arthritis, osteoarthritis, malignancy, history of joint arthroplasty, dental procedures, steroid use, previous surgery, and previous PJI |
| √ | Type of study designs used | Longitudinal studies (prospective or retrospective case control, prospective cohort, retrospective cohort, case-cohort, nested-case control, or clinical trials) |
| √ | Study population | Patients with at least one year follow-up for infection outcomes after total joint arthroplasty |
| **Reporting of search strategy should include** | |  |
| √ | Qualifications of searchers | Setor Kunutsor, PhD; Andrew Beswick, BSc |
| √ | Search strategy, including time period included in the synthesis and keywords | Time period: from inception to September, 2015.  The detailed search strategy can be found in Appendix D. |
| √ | Databases and registries searched | MEDLINE, EMBASE, Web of Science, and Cochrane databases |
| √ | Search software used, name and version, including special features | OvidSP was used to search EMBASE and MEDLINE  EndNote used to manage references |
| √ | Use of hand searching | We searched bibliographies of retrieved papers |
| √ | List of citations located and those excluded, including justifications | Details of the literature search process are outlined in the flow chart. The citation list for excluded studies is in Appendix Supplement 4. |
| √ | Method of addressing articles published in languages other than English | We placed no restrictions on language |
| √ | Method of handling abstracts and unpublished studies | We contacted several investigators for unpublished data and abstracts on the associations |
| √ | Description of any contact with authors | We contacted authors of studies that did not provide adequate data for analysis |
| **Reporting of methods should include** | |  |
| √ | Description of relevance or appropriateness of studies assembled for assessing the hypothesis to be tested | Detailed inclusion and exclusion criteria are described in the Methods section. |
| √ | Rationale for the selection and coding of data | Data extracted from each of the studies were relevant to the population characteristics, study design, exposure, and outcome. |
| √ | Assessment of confounding | We assessed confounding by ranking individual studies on the basis of different adjustment levels, and performed sub-group analyses to evaluate differences in the overall estimates according to levels of adjustment. |
| √ | Assessment of study quality, including blinding of quality assessors; stratification or regression on possible predictors of study results | Study quality was assessed based on the nine-star Newcastle–Ottawa Scale using pre-defined criteria namely: population representativeness, comparability (adjustment of confounders), ascertainment of outcome. Sensitivity analyses by several quality indicators such as study size, duration of follow-up, and adjustment factors. |
| √ | Assessment of heterogeneity | Heterogeneity of the studies was quantified with I^2^ statistic that provides the relative amount of variance of the summary effect due to the between-study heterogeneity and explored using meta-regression and stratified analyses |
| √ | Description of statistical methods in sufficient detail to be replicated | Description of methods of meta-analyses, sensitivity analyses, meta-regression and assessment of publication bias are detailed in the methods. We performed random effects meta-analysis with Stata 13. |
| √ | Provision of appropriate tables and graphics | Table 1 and Table A; Figs. 1-6; Figures A-M |
| **Reporting of results should include** | |  |
| √ | Graph summarizing individual study estimates and overall estimate | Figures A-K |
| √ | Table giving descriptive information for each study included | Table A |
| √ | Results of sensitivity testing | Sensitivity analysis was conducted to assess the influence of some large studies and low quality studies on the pooled estimate. This was done by omitting such studies and calculating a pooled estimate for the remainder of the studies |
| √ | Indication of statistical uncertainty of findings | 95% confidence intervals were presented with all summary estimates, I^2^ values and results of sensitivity analyses |
| **Reporting of discussion should include** | |  |
| √ | Quantitative assessment of bias | Sensitivity analyses indicate heterogeneity in strengths of the association due to most common biases in observational studies. The systematic review is limited in scope, as it involves published data. Individual participant data is needed. Limitations have been discussed. |
| √ | Justification for exclusion | All studies were excluded based on the pre-defined inclusion criteria in methods section. |
| √ | Assessment of quality of included studies | Brief discussion included in ‘Methods’ section |
| **Reporting of conclusions should include** | |  |
| √ | Consideration of alternative explanations for observed results | Discussion |
| √ | Generalization of the conclusions | Discussed in the context of the results. |
| √ | Guidelines for future research | We recommend analyses of individual participant data |
| √ | Disclosure of funding source | In “Acknowledgement” section |

**Appendix C.** Literature search strategy

Relevant studies, published from inception to September 1, 2015 (date last searched), were identified through electronic searches not limited to the English language using MEDLINE, EMBASE, Web of Science, and Cochrane databases. Electronic searches were supplemented by scanning reference lists of articles identified for all relevant studies (including review articles), by hand searching of relevant journals and by correspondence with study investigators. The computer-based searches combined search terms related to hip replacement, infection, and revision with focus on one- and two stage surgeries without language restriction.

1 prosthetic joint infection.mp. or exp Surgical Wound Infection/ (30119)

2 prosthetic infection.mp. (294)

3 wound infection.mp. or exp Wound Infection/ (45531)

4 exp Sepsis/ or sepsis.mp. (132792)

5 exp Surgical Wound Infection/ or surgical site infection.mp. (30357)

6 arthroplasty.mp. or exp Arthroplasty, Replacement, Knee/ or exp Arthroplasty, Replacement/ or exp Arthroplasty/ or exp Arthroplasty, Replacement, Hip/ (54094)

7 joint replacement.mp. (4292)

8 exp Joint Prosthesis/ or joint arthroplasty.mp. (37545)

9 exp Arthroplasty, Replacement, Hip/ or total arthroplasty.mp. (18367)

10 risk factor.mp. or exp Risk Factors/ (661103)

11 exp Biological Markers/ or exp Risk Factors/ or risk marker.mp. or exp Risk/ or exp Diabetes Mellitus, Type 2/ (1584727)

12 predictor.mp. (109243)

13 age.mp. (6925632)

14 sex.mp. or exp Sex/ (603694)

15 body mass index.mp. or exp Body Mass Index/ (142034)

16 body weight.mp. or exp Body Weight/ (451315)

17 socioeconomic status.mp. or exp Social Class/ (51072)

18 exp Smoking/ or smoking.mp. (202595)

19 exp "Tobacco Use"/ or exp Tobacco/ or tobacco.mp. (178015)

20 alcohol.mp. (214043)

21 diabetes.mp. or exp Diabetes Mellitus, Type 2/ or exp Diabetes Mellitus/ (438869)

22 exp Hypertension/ or hypertension.mp. (365828)

23 comorbidity.mp. or exp Comorbidity/ (91604)

24 rheumatoid arthritis.mp. or exp Arthritis, Rheumatoid/ (114884)

25 osteoarthritis.mp. or exp Osteoarthritis, Hip/ or exp Osteoarthritis/ or exp Osteoarthritis, Knee/ (55616)

26 history of joint arthroplasty.mp. (5)

27 dental procedure.mp. or exp Tooth Extraction/ (17354)

28 steroid.mp. or exp Steroids/ (779238)

29 anticoagulant.mp. or exp Anticoagulants/ (192581)

30 thromboprophylaxis.mp. (2762)

31 previous surgery.mp. (3074)

32 exp Femoral Fractures/ or exp Hip Fractures/ or exp Femoral Neck Fractures/ or previous fracture surgery.mp. (30456)

33 revision arthroplasty.mp. (973)

34 previous prosthetic joint infection.mp. (1)

35 1 or 2 or 3 or 4 or 5 (175563)

36 6 or 7 or 8 or 9 (70471)

37 10 or 11 or 12 or 13 or 14 or 15 or 16 or 17 or 18 or 19 or 20 or 21 or 22 or 23 or 24 or 25 or 26 or 27 or 28 or 29 or 30 or 31 or 32 or 33 or 34 (9860110)

38 35 and 36 (3173)

39 37 and 38 (2441)

40 limit 39 to humans (2421)

Each part was specifically translated for searching the other databases (EMBASE, Web of Science, and Cochrane databases)

**Appendix D.** Reference list of excluded studies

1. Padegimas EM, Maltenfort M, Ramsey ML, Williams GR, Parvizi J, Namdari S. Periprosthetic shoulder infection in the United States: Incidence and economic burden. *Journal of Shoulder and Elbow Surgery.* 2015;24(5):741-746.

2. Pope D, Scaife SL, Tzeng TH, Vasdev S, Saleh KJ. Impact of diabetes on early postoperative outcomes after total elbow arthroplasty. *Journal of Shoulder and Elbow Surgery.* 2015;24(3):348-352.

3. Singh JA, Inacio MCS, Namba RS, Paxton EW. Rheumatoid arthritis is associated with higher ninety-day hospital readmission rates compared to osteoarthritis after hip or knee arthroplasty: A cohort study. *Arthritis Care and Research.* 2015;67(5):718-724.

4. Stavrakis AI, SooHoo NF, Lieberman JR. A comparison of the incidence of complications following total hip arthroplasty in patients with or without osteonecrosis. *Journal of Arthroplasty.* 2015;30(1):114-117.

5. Tornero E, Garci-Ramiro S, Martinez-Pastor JC, et al. Prophylaxis with teicoplanin and cefuroxime reduces the rate of prosthetic joint infection after primary arthroplasty. *Antimicrobial Agents and Chemotherapy.* 2015;59(2):831-837.

6. Zywiel MG, Perruccio AV, Jackson T, Gandhi R. Comorbidities associated with end-stage knee osteoarthritis explain differences in the probability of major complications within 30 days of joint arthroplasty when compared to patients with hip osteoarthritis. *Osteoarthritis and Cartilage.* 2014;22:S380.

7. Wu JWS, Wong YC. Elective unilateral total knee replacement using continuous femoral nerve blockade versus conventional patient-controlled analgesia: Perioperative patient management based on a multidisciplinary pathway. *Hong Kong Medical Journal.* 2014;20(1):45-51.

8. Wang Z, Anderson FA, Jr., Ward M, Bhattacharyya T. Surgical site infections and other postoperative complications following prophylactic anticoagulation in total joint arthroplasty. *PLoS ONE [Electronic Resource].* 2014;9(4):e91755.

9. Pruzansky JS, Bronson MJ, Grelsamer RP, Strauss E, Moucha CS. Prevalence of modifiable surgical site infection risk factors in hip and knee joint arthroplasty patients at an urban academic hospital. *Journal of Arthroplasty.* 2014;29(2):272-276.

10. Carroll K, Dowsey M, Choong P, Peel T. Risk factors for superficial wound complications in hip and knee arthroplasty. *Clinical Microbiology & Infection.* 2014;20(2):130-135.

11. Singh JA, Hawn M, Campagna EJ, Henderson WG, Richman J, Houston TK. Mediation of smoking-associated postoperative mortality by perioperative complications in veterans undergoing elective surgery: Data from Veterans Affairs Surgical Quality Improvement Program (VASQIP)a cohort study. *BMJ Open.* 2013;3(4).

12. Rasouli MR, Maltenfort MG, Purtill JJ, Hozack WJ, Parvizi J. Has the rate of in-hospital infections after total joint arthroplasty decreased? *Clinical Orthopaedics & Related Research.* 2013;471(10):3102-3111.

13. Poultsides LA, Ma Y, Della Valle AG, Chiu YL, Sculco TP, Memtsoudis SG. In-hospital surgical site infections after primary hip and knee arthroplasty--incidence and risk factors. *Journal of Arthroplasty.* 2013;28(3):385-389.

14. Griffin JW, Novicoff WM, Browne JA, Brockmeier SF. Obstructive sleep apnea as a risk factor after shoulder arthroplasty. *Journal of Shoulder and Elbow Surgery.* 2013;22(12):e6-e9.

15. Easterlin MC, Chang DG, Talamini M, Chang DC. Older age increases short-term surgical complications after primary knee arthroplasty knee. *Clinical Orthopaedics and Related Research.* 2013;471(8):2611-2620.

16. Aggarwal VK, Tischler EH, Post ZD, Kane I, Orozco FR, Ong A. Patients with atrial fibrillation undergoing total joint arthroplasty increase hospital burden. *Journal of Bone and Joint Surgery - Series A.* 2013;95(17):1606-1611.

17. Masgala A, Chronopoulos E, Nikolopoulos G, et al. Risk factors affecting the incidence of infection after orthopaedic surgery: the role of chemoprophylaxis. *Central European Journal of Public Health.* 2012;20(4):252-256.

18. Skaar DD, O'Connor H, Hodges JS, Michalowicz BS. Dental procedures and subsequent prosthetic joint infections: findings from the Medicare Current Beneficiary Survey. *Journal of the American Dental Association.* 2011;142(12):1343-1351.

19. Font-Vizcarra L, Tornero E, Bori G, Bosch J, Mensa J, Soriano A. Relationship between intraoperative cultures during hip arthroplasty, obesity, and the risk of early prosthetic joint infection: a prospective study of 428 patients. *International Journal of Artificial Organs.* 2011;34(9):870-875.

20. Asensio A, Antolin FJ, Sanchez-Garcia JM, et al. Timing of DVT prophylaxis and risk of postoperative knee prosthesis infection. *Orthopedics.* 2010;33(11):800.

21. Pfitzner T, Krocker D, Perka C, Matziolis G. [C-reactive protein. An independent risk factor for the development of infection after primary arthroplasty]. *Orthopade.* 2008;37(11):1116-1120.

22. Horne G, Devane P, Davidson A, Adams K, Purdie G. The influence of steroid injections on the incidence of infection following total knee arthroplasty. *New Zealand Medical Journal.* 2008;121(1268):U2896.

23. Gunningberg L, Persson C, Akerfeldt T, Stridsberg M, Swenne CL. Pre- and postoperative nutritional status and predictors for surgical-wound infections in elective orthopaedic and thoracic patients. *e-SPEN.* 2008;3(3):e93-e101.

24. Jover-Saenz A, Barcenilla-Gaite F, Torres-Puig-Gros J, Prats-Gispert L, Garrido-Calvo S, Porcel-Perez JM. [Risk factors for total prosthetic joint infection. Case-control study]. *Medicina Clinica.* 2007;128(13):493-494.

25. Ridgeway S, Wilson J, Charlet A, Kafatos G, Pearson A, Coello R. Infection of the surgical site after arthroplasty of the hip. *Journal of Bone & Joint Surgery - British Volume.* 2005;87(6):844-850.

26. Mahomed NN, Barrett JA, Katz JN, et al. Rates and outcomes of primary and revision total hip replacement in the United States medicare population. *Journal of Bone & Joint Surgery - American Volume.* 2003;85-A(1):27-32.

27. Saleh K, Olson M, Resig S, et al. Predictors of wound infection in hip and knee joint replacement: results from a 20 year surveillance program. *Journal of Orthopaedic Research.* 2002;20(3):506-515.

28. Beck-Sague CM, Chong WH, Roy C, Anderson R, Jarvis WR. Outbreak of surgical wound infections associated with total hip arthroplasty. *Infection Control & Hospital Epidemiology.* 1992;13(9):526-534.

29. Peersman G, Laskin R, Davis J, Peterson MG, Richart T. ASA physical status classification is not a good predictor of infection for total knee replacement and is influenced by the presence of comorbidities. *Acta Orthopaedica Belgica.* 2008;74(3):360-364.

30. Cumming D, Parker MJ. Urinary catheterisation and deep wound infection after hip fracture surgery. *International Orthopaedics.* 2007;31(4):483-485.

31. Iorio R, Williams KM, Marcantonio AJ, Specht LM, Tilzey JF, Healy WL. Diabetes mellitus, hemoglobin A1C, and the incidence of total joint arthroplasty infection. *J Arthroplasty.* 2012;27(5):726-729 e721.

32. Smith C, Christiansen T, Khong H, Werle J. Association between obesity and inpatient adverse events following primary hip or knee arthroplasty. *Osteoarthritis and Cartilage.* 2015;23:A343.

33. Smucny M, Menendez ME, Ring D, Feeley BT, Zhang AL. Inpatient surgical site infection after shoulder arthroplasty. *Journal of Shoulder and Elbow Surgery.* 2015;24(5):747-753.

34. Napier RJ, O'Brien S, Bennett D, et al. Intra-operative and short term outcome of total knee arthroplasty in morbidly obese patients. *Knee.* 2014;21(3):784-788.

35. Danninger T, Rasul R, Poeran J, et al. Blood transfusions in total hip and knee arthroplasty: An analysis of outcomes. *The Scientific World Journal.* 2014;2014(623460).

36. Hsieh PH, Huang KC, Shih HN. Prosthetic joint infection in patients with rheumatoid arthritis: an outcome analysis compared with controls. *PLoS ONE [Electronic Resource].* 2013;8(8):e71666.

37. Suleiman LI, Ortega G, Ong'Uti SK, et al. Does BMI affect perioperative complications following total knee and hip arthroplasty? *Journal of Surgical Research.* 2012;174(1):7-11.

38. Mraovic B, Jacovides C, Joseph J, Parvizi J. Postoperative morning hyperglycemia increases risk for periprosthetic joint infection after HIP and knee arthroplasty. *Regional Anesthesia and Pain Medicine.* 2011;36 (5).

39. Memtsoudis SG, Ma Y, Chiu YL, Poultsides L, Gonzalez Della Valle A, Mazumdar M. Bilateral total knee arthroplasty: Risk factors for major morbidity and mortality. *Anesthesia and Analgesia.* 2011;113(4):784-790.

40. Jamsen E, Huhtala H, Puolakka T, Moilanen T. Risk factors for infection after knee arthroplasty. A register-based analysis of 43,149 cases. *The Journal of bone and joint surgery. American volume.* 2009;91(1):38-47.

41. Mortazavi SM, Schwartzenberger J, Austin MS, Purtill JJ, Parvizi J. Revision total knee arthroplasty infection: incidence and predictors. *Clinical Orthopaedics & Related Research.* 2010;468(8):2052-2059.

42. Edwards C, Counsell A, Boulton C, Moran CG. Early infection after hip fracture surgery: risk factors, costs and outcome. *Journal of Bone & Joint Surgery - British Volume.* 2008;90(6):770-777.

43. Wymenga AB, van Horn JR, Theeuwes A, Muytjens HL, Slooff TJ. Perioperative factors associated with septic arthritis after arthroplasty. Prospective multicenter study of 362 knee and 2,651 hip operations. *Acta Orthopaedica Scandinavica.* 1992;63(6):665-671.

44. Collins DN, McKenzie JM. Infections at the site of a hip implant: Successful and unsuccessful management. *Clinical Orthopaedics and Related Research.* 1991(269):9-15.

45. Miric A, Lim M, Kahn B, Rozenthal T, Bombick D, Sculco TP. Perioperative morbidity following total knee arthroplasty among obese patients. *J Knee Surg.* 2002;15(2):77-83.

46. Grant JA, Viens N, Bolognesi MP, Olson SA, Cook CE. Two-year outcomes in primary THA in obese male veterans administration medical center patients. *Rheumatology International.* 2008;28(11):1105-1109.

47. Bolognesi MP, Marchant MH, Jr., Viens NA, Cook C, Pietrobon R, Vail TP. The impact of diabetes on perioperative patient outcomes after total hip and total knee arthroplasty in the United States. *J Arthroplasty.* 2008;23(6 Suppl 1):92-98.

48. Sathiyakumar V, Greenberg SE, Molina CS, Thakore RV, Obremskey WT, Sethi MK. Hip fractures are risky business: An analysis of the NSQIP data. *Injury.* 2015;46(4):703-708.

49. Minnema B, Vearncombe M, Augustin A, Gollish J, Simor AE. Risk factors for surgical-site infection following primary total knee arthroplasty. *Infection Control & Hospital Epidemiology.* 2004;25(6):477-480.

50. Castella A, Argentero PA, Farina EC, Charrier L, Del Prever EMB, Zotti CM. Incidence of surgical-site infections in orthopaedic surgery: A northern Italian experience. *Epidemiology and Infection.* 2011;139(5):777-782.

51. Jamsen E, Nevalainen P, Kalliovalkama J, Moilanen T. Preoperative hyperglycemia predicts infected total knee replacement. *European Journal of Internal Medicine.* 2010;21(3):196-201.

52. Darwish FM, Haddad WH, Aloudat Z, Aboharfil A. Total knee replacement in King Abdullah University Hospital, early results. *Jordan Medical Journal.* 2009;43(3):163-170.

53. Clements ACA, Tong ENC, Morton AP, Whitby M. Risk stratification for surgical site infections in Australia: evaluation of the US National Nosocomial Infection Surveillance risk index. *Journal of Hospital Infection.* 2007;66(2):148-155.

54. Thomas C, Cadwallader HL, Riley TV. Surgical-site infections after orthopaedic surgery: statewide surveillance using linked administrative databases. *Journal of Hospital Infection.* 2004;57(1):25-30.

55. Waldman BJ, Mont MA, Hungerford DS. Total knee arthroplasty infections associated with dental procedures. *Clinical Orthopaedics & Related Research.* 1997(343):164-172.

56. Luessenhop CP, Higgins LD, Brause BD, Ranawat CS. Multiple prosthetic infections after total joint arthroplasty. Risk factor analysis. *Journal of Arthroplasty.* 1996;11(7):862-868.

57. Gordon SM, Culver DH, Simmons BP, Jarvis WR. Risk factors for wound infections after total knee arthroplasty. *American Journal of Epidemiology.* 1990;131(5):905-916.

58. Tomas T. [Patient - related risk factors for infected total arthroplasty]. *Acta Chirurgiae Orthopaedicae et Traumatologiae Cechoslovaca.* 2008;75(6):451-456.

59. Syahrizal AB, Kareem BA, Anbanadan S, Harwant S. Risk factors for infection in total knee replacement surgery at hospital Kuala Lumpur. *Medical Journal of Malaysia.* 2001;56 Suppl D:5-8.

60. Lazzarini L, Pellizzer G, Stecca C, Viola R, de Lalla F. Postoperative infections following total knee replacement: an epidemiological study. *Journal of Chemotherapy.* 2001;13(2):182-187.

61. Wolfe SW, Figgie MP, Inglis AE, Bohn WW, Ranawat CS. Management of infection about total elbow prostheses. *Journal of Bone & Joint Surgery - American Volume.* 1990;72(2):198-212.

62. Morrey BF, Bryan RS. Infection after total elbow arthroplasty. *Journal of Bone & Joint Surgery - American Volume.* 1983;65(3):330-338.

63. Lau ACK, Neo GH, Lee HC. Risk factors of surgical site infections in hip hemiarthroplasty: A single-institution experience over nine years. *Singapore Medical Journal.* 2014;55(10):535-538.

64. McCalden RW, Charron KD, MacDonald SJ, Bourne RB, Naudie DD. Does morbid obesity affect the outcome of total hip replacement?: an analysis of 3290 THRs. *Journal of Bone & Joint Surgery - British Volume.* 2011;93(3):321-325.

65. Thu LT, Dibley MJ, Ewald B, Tien NP, Lam LD. Incidence of surgical site infections and accompanying risk factors in Vietnamese orthopaedic patients. *J Hosp Infect.* 2005;60(4):360-367.

66. Garcia-Alvarez F, Al-Ghanem R, Garcia-Alvarez I, Lopez-Baisson A, Bernal M. Risk factors for postoperative infections in patients with hip fracture treated by means of Thompson arthroplasty. *Archives of Gerontology & Geriatrics.* 2010;50(1):51-55.

67. Lee J, Singletary R, Schmader K, Anderson DJ, Bolognesi M, Kaye KS. Surgical site infection in the elderly following orthopaedic surgery. Risk factors and outcomes. *Journal of Bone & Joint Surgery - American Volume.* 2006;88(8):1705-1712.

**Appendix E.** Reference list of included studies

1. Surin VV, Sundholm K, Backman L. Infection after total hip replacement. With special reference to a discharge from the wound. *Journal of Bone & Joint Surgery - British Volume.* 1983;65(4):412-418.

2. Vannini P, Ciavarella A, Olmi R, et al. Diabetes as pro-infective risk factor in total hip replacement. *Acta diabetologica latina.* 1984;21(3):275-280.

3. Wilson MG, Kelley K, Thornhill TS. Infection as a complication of total knee-replacement arthroplasty. Risk factors and treatment in sixty-seven cases. *J Bone Joint Surg Am.* 1990;72(6):878-883.

4. Bengtson S, Knutson K. The infected knee arthroplasty. A 6-year follow-up of 357 cases. *Acta orthopaedica Scandinavica.* 1991;62(4):301-311.

5. Papagelopoulos PJ, Idusuyi OB, Wallrichs SL, Morrey BF. Long term outcome and survivorship analysis of primary total knee arthroplasty in patients with diabetes mellitus. *Clin Orthop Relat Res.* 1996(330):124-132.

6. Berbari EF, Hanssen AD, Duffy MC, et al. Risk factors for prosthetic joint infection: case-control study. *Clinical Infectious Diseases.* 1998;27(5):1247-1254.

7. de Boer AS, Geubbels EL, Wille J, Mintjes-de Groot AJ. Risk assessment for surgical site infections following total hip and total knee prostheses. *Journal of Chemotherapy.* 2001;13 Spec No 1(1):42-47.

8. Yong KS, Kareem BA, Ruslan GN, Harwant S. Risk factors for infection in total hip replacement surgery at Hospital Kuala Lumpur. *Medical Journal of Malaysia.* 2001;56 Suppl C:57-60.

9. Meding JB, Reddleman K, Keating ME, et al. Total knee replacement in patients with diabetes mellitus. *Clin Orthop Relat Res.* 2003(416):208-216.

10. Foran JR, Mont MA, Rajadhyaksha AD, Jones LC, Etienne G, Hungerford DS. Total knee arthroplasty in obese patients: a comparison with a matched control group. *J Arthroplasty.* 2004;19(7):817-824.

11. Namba RS, Paxton L, Fithian DC, Stone ML. Obesity and perioperative morbidity in total hip and total knee arthroplasty patients. *J Arthroplasty.* 2005;20(7 Suppl 3):46-50.

12. Amin AK, Clayton RAE, Patton JT, Gaston M, Cook RE, Brenkel IJ. Total knee replacement in morbidly obese patients: Results of a prospective, matched study. *Journal of Bone and Joint Surgery - Series B.* 2006;88(10):1321-1326.

13. Amin AK, Patton JT, Cook RE, Brenkel IJ. Does obesity influence the clinical outcome at five years following total knee replacement for osteoarthritis? *Journal of Bone & Joint Surgery - British Volume.* 2006;88(3):335-340.

14. Garcia-Pont J, Blanch-Falp J, Coll-Colell R, et al. [Prosthetic joint infection: a prospective study in five Catalonian hospitals]. *Enfermedades Infecciosas y Microbiologia Clinica.* 2006;24(3):157-161.

15. McIntosh AL, Hanssen AD, Wenger DE, Osmon DR. Recent intraarticular steroid injection may increase infection rates in primary THA. *Clinical Orthopaedics & Related Research.* 2006;451:50-54.

16. Muilwijk J, Walenkamp GH, Voss A, Wille JC, van den Hof S. Random effect modelling of patient-related risk factors in orthopaedic procedures: results from the Dutch nosocomial infection surveillance network 'PREZIES'. *Journal of Hospital Infection.* 2006;62(3):319-326.

17. Papavasiliou AV, Isaac DL, Marimuthu R, Skyrme A, Armitage A. Infection in knee replacements after previous injection of intra-articular steroid. *Journal of Bone and Joint Surgery - Series B.* 2006;88(3):321-323.

18. Babkin Y, Raveh D, Lifschitz M, et al. Incidence and risk factors for surgical infection after total knee replacement. *Scandinavian Journal of Infectious Diseases.* 2007;39(10):890-895.

19. Choong PF, Dowsey MM, Carr D, Daffy J, Stanley P. Risk factors associated with acute hip prosthetic joint infections and outcome of treatment with a rifampinbased regimen. *Acta Orthopaedica.* 2007;78(6):755-765.

20. Huotari K, Lyytikainen O, Seitsalo S. Patient outcomes after simultaneous bilateral total hip and knee joint replacements. *Journal of Hospital Infection.* 2007;65(3):219-225.

21. Krushell RJ, Fingeroth RJ. Primary Total Knee Arthroplasty in Morbidly Obese Patients: a 5- to 14-year follow-up study. *J Arthroplasty.* 2007;22(6 Suppl 2):77-80.

22. Lai K, Bohm ER, Burnell C, Hedden DR. Presence of medical comorbidities in patients with infected primary hip or knee arthroplasties. *Journal of Arthroplasty.* 2007;22(5):651-656.

23. Lubbeke A, Stern R, Garavaglia G, Zurcher L, Hoffmeyer P. Differences in outcomes of obese women and men undergoing primary total hip arthroplasty. *Arthritis Rheum.* 2007;57(2):327-334.

24. Pasticci MB, Mancini G, Lapalorcia LM, et al. Prosthetic infections following total knee arthroplasty: A six-year prospective study (1997-2002). *Journal of Orthopaedics and Traumatology.* 2007;8(1):25-28.

25. van Kasteren ME, Mannien J, Ott A, Kullberg BJ, de Boer AS, Gyssens IC. Antibiotic prophylaxis and the risk of surgical site infections following total hip arthroplasty: timely administration is the most important factor. *Clinical Infectious Diseases.* 2007;44(7):921-927.

26. Bongartz T, Halligan CS, Osmon DR, et al. Incidence and risk factors of prosthetic joint infection after total hip or knee replacement in patients with rheumatoid arthritis. *Arthritis & Rheumatism.* 2008;59(12):1713-1720.

27. Chesney D, Sales J, Elton R, Brenkel IJ. Infection after knee arthroplasty a prospective study of 1509 cases. *Journal of Arthroplasty.* 2008;23(3):355-359.

28. Dowsey MM, Choong PF. Obesity is a major risk factor for prosthetic infection after primary hip arthroplasty. *Clinical Orthopaedics & Related Research.* 2008;466(1):153-158.

29. Fan JC, Hung HH, Fung KY. Infection in primary total knee replacement. *Hong Kong Medical Journal.* 2008;14(1):40-45.

30. Moon HK, Han CD, Yang IH, Cha BS. Factors affecting outcome after total knee arthroplasty in patients with diabetes mellitus. *Yonsei Medical Journal.* 2008;49(1):129-137.

31. Pulido L, Ghanem E, Joshi A, Purtill JJ, Parvizi J. Periprosthetic joint infection: the incidence, timing, and predisposing factors. *Clin Orthop Relat Res.* 2008;466(7):1710-1715.

32. Desai A, Ramankutty S, Board T, Raut V. Does intraarticular steroid infiltration increase the rate of infection in subsequent total knee replacements? *Knee.* 2009;16(4):262-264.

33. Dowsey MM, Choong PF. Obese diabetic patients are at substantial risk for deep infection after primary TKA. *Clinical Orthopaedics & Related Research.* 2009;467(6):1577-1581.

34. Ong KL, Kurtz SM, Lau E, Bozic KJ, Berry DJ, Parvizi J. Prosthetic joint infection risk after total hip arthroplasty in the Medicare population. *Journal of Arthroplasty.* 2009;24(6 Suppl):105-109.

35. Dowsey MM, Liew D, Stoney JD, Choong PF. The impact of pre-operative obesity on weight change and outcome in total knee replacement: a prospective study of 529 consecutive patients. *J Bone Joint Surg Br.* 2010;92(4):513-520.

36. Kurtz SM, Ong KL, Lau E, Bozic KJ, Berry D, Parvizi J. Prosthetic joint infection risk after TKA in the Medicare population. *Clinical Orthopaedics & Related Research.* 2010;468(1):52-56.

37. Malinzak RA, Ritter MA, Berend ME, Meding JB, Olberding EM, Davis KE. Morbidly obese, diabetic, younger, and unilateral joint arthroplasty patients have elevated total joint arthroplasty infection rates. *J Arthroplasty.* 2009;24(6 Suppl):84-88.

38. Aslam S, Reitman C, Darouiche RO. Risk factors for subsequent diagnosis of prosthetic joint infection. *Infection Control & Hospital Epidemiology.* 2010;31(3):298-301.

39. Berbari EF, Osmon DR, Carr A, et al. Dental procedures as risk factors for prosthetic hip or knee infection: a hospital-based prospective case-control study.[Erratum appears in Clin Infect Dis. 2010 Mar 15;50(6):944]. *Clinical Infectious Diseases.* 2010;50(1):8-16.

40. Chee YH, Teoh KH, Sabnis BM, Ballantyne JA, Brenkel IJ. Total hip replacement in morbidly obese patients with osteoarthritis: Results of a prospectively matched study. *Journal of Bone and Joint Surgery - Series B.* 2010;92(8):1066-1071.

41. Cordero-Ampuero J, de Dios M. What are the risk factors for infection in hemiarthroplasties and total hip arthroplasties? *Clin Orthop Relat Res.* 2010;468(12):3268-3277.

42. Levent T, Vandevelde D, Delobelle JM, et al. Infection risk prevention following total knee arthroplasty. *Orthopaedics & traumatology, surgery & research.* 2010;96(1):49-56.

43. Willis-Owen CA, Konyves A, Martin DK. Factors affecting the incidence of infection in hip and knee replacement: an analysis of 5277 cases. *Journal of Bone & Joint Surgery - British Volume.* 2010;92(8):1128-1133.

44. Dale H, Skramm I, Lower HL, et al. Infection after primary hip arthroplasty: a comparison of 3 Norwegian health registers. *Acta Orthopaedica.* 2011;82(6):646-654.

45. Davis AM, Wood AM, Keenan ACM, Brenkel IJ, Ballantyne JA. Does body mass index affect clinical outcome post-operatively and at five years after primary unilateral total hip replacement performed for osteoarthritis?: A multivariate analysis of prospective data. *Journal of Bone and Joint Surgery - Series B.* 2011;93 B(9):1178-1182.

46. Peel TN, Dowsey MM, Daffy JR, Stanley PA, Choong PF, Buising KL. Risk factors for prosthetic hip and knee infections according to arthroplasty site. *J Hosp Infect.* 2011;79(2):129-133.

47. Suzuki G, Saito S, Ishii T, Motojima S, Tokuhashi Y, Ryu J. Previous fracture surgery is a major risk factor of infection after total knee arthroplasty. *Knee surgery, sports traumatology, arthroscopy : official journal of the ESSKA.* 2011;19(12):2040-2044.

48. Ayyar V, Burnett R, Coutts FJ, Van Der Linden ML, Mercer TH. The influence of obesity on patient reported outcomes following total knee replacement. *Arthritis.* 2012;1(1).

49. Bozic KJ, Lau E, Kurtz S, et al. Patient-related risk factors for periprosthetic joint infection and postoperative mortality following total hip arthroplasty in Medicare patients. *Journal of Bone & Joint Surgery - American Volume.* 2012;94(9):794-800.

50. Kessler B, Sendi P, Graber P, et al. Risk factors for periprosthetic ankle joint infection: a case-control study. *J Bone Joint Surg Am.* 2012;94(20):1871-1876.

51. Meermans G, Corten K, Simon JP. Is the infection rate in primary THA increased after steroid injection? *Clin Orthop.* 2012;470(11):3213-3219.

52. Namba RS, Inacio MC, Paxton EW. Risk factors associated with surgical site infection in 30,491 primary total hip replacements. *Journal of Bone & Joint Surgery - British Volume.* 2012;94(10):1330-1338.

53. Singh JA, Sperling JW, Schleck C, Harmsen WS, Cofield RH. Periprosthetic infections after total shoulder arthroplasty: a 33-year perspective. *Journal of shoulder and elbow surgery / American Shoulder and Elbow Surgeons ... [et al.].* 2012;21(11):1534-1541.

54. Song KH, Kim ES, Kim YK, et al. Differences in the risk factors for surgical site infection between total hip arthroplasty and total knee arthroplasty in the Korean Nosocomial Infections Surveillance System (KONIS). *Infect Control Hosp Epidemiol.* 2012;33(11):1086-1093.

55. Jamsen E, Nevalainen P, Eskelinen A, Huotari K, Kalliovalkama J, Moilanen T. Obesity, diabetes, and preoperative hyperglycemia as predictors of periprosthetic joint infection: a single-center analysis of 7181 primary hip and knee replacements for osteoarthritis. *Journal of Bone & Joint Surgery - American Volume.* 2012;94(14):e101.

56. Adams AL, Paxton EW, Wang JQ, et al. Surgical outcomes of total knee replacement according to diabetes status and glycemic control, 2001 to 2009. *Journal of Bone & Joint Surgery - American Volume.* 2013;95(6):481-487.

57. Durand F, Berthelot P, Cazorla C, Farizon F, Lucht F. Smoking is a risk factor of organ/space surgical site infection in orthopaedic surgery with implant materials. *Int Orthop.* 2013;37(4):723-727.

58. Everhart JS, Altneu E, Calhoun JH. Medical comorbidities are independent preoperative risk factors for surgical infection after total joint arthroplasty. *Clin Orthop.* 2013;471(10):3112-3119.

59. Namba RS, Inacio MC, Paxton EW. Risk factors associated with deep surgical site infections after primary total knee arthroplasty: an analysis of 56,216 knees. *Journal of Bone & Joint Surgery - American Volume.* 2013;95(9):775-782.

60. Bozic KJ, Lau E, Ong K, et al. Risk factors for early revision after primary total hip arthroplasty in Medicare patients. *Clinical Orthopaedics & Related Research.* 2014;472(2):449-454.

61. Gou W, Chen J, Jia Y, Wang Y. Preoperative asymptomatic leucocyturia and early prosthetic joint infections in patients undergoing joint arthroplasty. *J Arthroplasty.* 2014;29(3):473-476.

62. Ravi B, Croxford R, Hollands S, et al. Increased risk of complications following total joint arthroplasty in patients with rheumatoid arthritis. *Arthritis & rheumatology.* 2014;66(2):254-263.

63. Rasouli MR, Restrepo C, Maltenfort MG, Purtill JJ, Parvizi J. Risk factors for surgical site infection following total joint arthroplasty. *J Bone Joint Surg Am.* 2014;96(18):e158.

64. Sousa R, Munoz-Mahamud E, Quayle J, et al. Is asymptomatic bacteriuria a risk factor for prosthetic joint infection? *Clinical infectious diseases : an official publication of the Infectious Diseases Society of America.* 2014;59(1):41-47.

65. Tai SM, Imbuldeniya AM, Munir S, Walter WL, Walter WK, Zicat BA. The effect of obesity on the clinical, functional and radiological outcome of cementless total hip replacement: a case-matched study with a minimum 10-year follow-up. *J Arthroplasty.* 2014;29(9):1758-1762.

66. Maoz G, Phillips M, Bosco J, et al. The Otto Aufranc Award: Modifiable versus nonmodifiable risk factors for infection after hip arthroplasty. *Clin Orthop.* 2015;473(2):453-459.

**Table A.** Characteristics of studies included in review

| **Lead Author, Publication Date (Reference No.)** | **Location** | **Baseline year of study** | **Mean /median age (years)** | **% male** | **Follow up**  **Mean/median (year)** | **Type of arthroplasty** | **No. of participants or arthroplasties** | **No. of infection cases** | **Quality score** |
| --- | --- | --- | --- | --- | --- | --- | --- | --- | --- |
|  |  |  |  |  |  |  |  |  |  |
| Surin, 1983 | Sweden | 1970-1977 | 64.0 | NR | 3-10 | THA | 803 | 34 | 5 |
| Vannini, 1984 | Italy | 1969-1979 | 62.1 | 30.2 | 2.9 | THA | 1,042 | 28 | 5 |
| Wilson, 1990 | USA | 1973-1987 | NR | NR | 7.0 | TKA | 4,171 | 67 | 5 |
| Bengston, 1991 | Sweden | 1975-1985 | 69.0 | 28.1 | 6.0 | TKA | 12,118 | 309 | 5 |
| Papagelopoulos, 1996 | USA | 1978-1982 | NR | 49.0 | 8.0 | TKA | 136 | 1 | 5 |
| Berbari, 1998 | USA | 1969-1991 | 61.7 | 50.0 | 1.4 | TKA and THA | 924 | 462 | 5 |
| de Boer, 2001 | Netherlands | 1996-1999 | NR | NR | 1.0 | TKA and THA | 16,790 | 133 | 6 |
| Yong, 2001 | Malaysia | 1992-1998 | NR | NR | 3.3 | THA | 67 | 8 | 6 |
| Meding, 2003 | USA | 1987-1999 | 70.0 | 40.7 | 4.3 | TKA | 3,519 | 39 | 5 |
| Foran, 2004 | USA | 1991-1996 | 68.0 | 32.4 | 6.7 | TKA | 136 | 2 | 6 |
| Namba, 2005 | USA | 2001-2002 | 66.9 | 39.9 | 1.0 | TKA and THA | 2,884 | 10 | 5 |
| Amin, 2006 (a) | UK | 1995-2004 | 62.0 | 26.8 | 3.2 | TKA | 82 | 2 | 5 |
| Amin, 2006 (b) | UK | 1995-1997 | 68.7 | 42.4 | 5.0 | TKA | 370 | 3 | 5 |
| Garcia-Pont, 2006 | Spain | 2001 | 71.0 | 26.9 | 2.0 | TKA and THA | 425 | 14 | 7 |
| McIntosh, 2006 | USA | 1998-2002 | 70.0 | 42.3 | 2.7 | THA | 448 | 4 | 6 |
| Muilwijk, 2006 | Netherlands | 1996-2003 | NR | NR | 1.0 | THA | 26,127 | 493 | 7 |
| Papavasiliou, 2006 | UK | 2002-2004 | NR | NR | 2.5 | TKA | 144 | 3 | 5 |
| Babkin, 2007 | Israel | 1999-2000 | 72.4 | 33.9 | 1.0 | TKA | 180 | 10 | 5 |
| Choong, 2007 | Australia | 1998-2004 | 68.1 | 46.0 | 3.5 | THA | 819 | 14 | 5 |
| Huotari, 2007 | Finland | 2001-2004 | 64.8 | 40.5 | 1.0 | TKA and THA | 9,831 | 61 | 5 |
| Krushell, 2007 | USA | 1992-1999 | 68.2 | 7.7 | 7.5 | TKA | 78 | 8 | 5 |
| Lai, 2007 | Canada | NR | 62.5 | 52.9 | Up to 17 | TKA and THA | 102 | 52 | 5 |
| Lubbeke, 2007 | Switzerland | 1996-2005 | 68.6 | 44.6 | 5.0 | THA | 2,495 | 17 | 7 |
| Pasticci, 2007 | Italy | 1997-2002 | 71.0 | 37.0 | 2.0 | TKA | 171 | 3 | 6 |
| van Kasteren, 2007 | Netherlands | 2000-2002 | 68.8 | 31.0 | 1.0 | THA | 1,922 | 50 | 7 |
| Bongartz, 2008 | USA | 1996-2004 | 64.0 | 21.4 | 4.3 | TKA and THA | 657 | 23 | 5 |
| Chesney, 2008 | UK | 1998-2005 | NR | 47.7 | 5.0 | TKA | 1,509 | 16 | 7 |
| Dowsey, 2008 | Australia | 1998-2005 | 69.0 | 44.2 | 1.0 | THA | 1,207 | 22 | 5 |
| Fan, 2008 | Hong Kong | 1997-2006 | 69.0 | 82.4 | 3.8 | TKA | 472 | 14 | 5 |
| Moon, 2008 | Korea | 1995-2004 | 67.5 | 9.4 | 4.4 | TKA | 444 | 3 | 5 |
| Pulido, 2008 | USA | 2001-2006 | 64.3 | 42.0 | 3.6 | TKA and THA | 9,245 | 63 | 6 |
| Desai, 2009 | UK | 1997-2005 | 70.7 | 37.0 | 3.6 | THA | 270 | 7 | 5 |
| Dowsey, 2009 | Australia | 1998-2005 | 72.0 | 37.0 | 1.0 | TKA | 1,214 | 18 | 7 |
| Ong, 2009 | USA | 1997-2006 | NR | NR | 10.0 | THA | 39,929 | 887 | 7 |
| Dowsey, 2010 | Australia | 2006-2007 | 71.4 | 31.0 | 1.0 | TKA | 529 | 8 | 7 |
| Kurtz, 2010 | USA | 1997-2006 | NR | NR | 2-10 | TKA | 69,663 | 1,400 | 7 |
| Malinzak, 2009 | USA | 1991-2004 | 68.8 | 40.7 | 6.2 | TKA and THA | 8,494 | 43 | 6 |
| Aslam, 2010 | USA | 1997-2006 | 60.8 | 85.7 | 2.9 | TKA, THA, and TSA | 126 | 63 | 5 |
| Berbari, 2010 | USA | 2001-2006 | 70.5 | 48.7 | 2.0 | TKA and THA | 678 | 339 | 7 |
| Chee, 2010 | UK | 1998-2003 | 64.0 | 22.6 | 5.0 | THA | 110 | 2 | 5 |
| Cordero-Ampuero, 2010 | Spain | 1997-2007 | 68.6 | 70.2 | 2.3 | THA | 124 | 24 | 5 |
| Levent, 2010 | France | 2005-2006 | 72.0 | 27.0 | 1.0 | TKA | 364 | 5 | 6 |
| Willis-Owen, 2010 | Australia | 2002-2008 | 65.6 | 49.3 | 1.0 | TKA and THA | 5,277 | 51 | 5 |
| Dale, 2011 | Norway | 2005-2009 | NR | NR | 1.0 | THA | 5,540 | 167 | 7 |
| Davis, 2011 | UK | 1998-2005 | 69.0 | 38.5 | 5.0 | THA | 1,617 | 83 | 8 |
| Peel, 2011 | Australia | 2000-2007 | 69.0 | NR | 1.0 | TKA and THA | 108 | 36 | 5 |
| Suzuki, 2011 | Japan | 1995-2006 | 72.0 | 22.0 | 3.5 | TKA | 1,146 | 17 | 5 |
| Ayyar, 2012 | UK | 2005-2008 | 66.7 | 39.7 | 1.0 | TKA | 171 | 16 | 5 |
| Bozic, 2012 | USA | 1998-2007 | NS | NS | Up to 10 | THA | 40,919 | 1,371 | 7 |
| Kessler, 2012 | Switzerland | 2006-2010 | 63.0 | 45.4 | Up to 10 | TAA | 130 | 26 | 5 |
| Meermans, 2012 | Belgium | 1998-2006 | 66.5 | 28.0 | 5.9 | THA | 364 | 2 | 5 |
| Namba, 2012 | USA | 2001-2009 | 65.5 | 43.0 | 1.0 | THA | 30,491 | 155 | 7 |
| Singh, 2012 | USA | 1976-2008 | 65.0 | 47.0 | 7.0 | TSA | 2,588 | 32 | 8 |
| Song, 2012 | Korea | 2006-2009 | 67.0 | 26.0 | 1.0 | TKA and THA | 6,848 | 161 | 7 |
| Jamsen, 2012 | Finland | 2002-2008 | 70.6 | 36.1 | 1.0 | TKA and THA | 7,181 | 52 | 7 |
| Adams, 2013 | USA | 2001-2009 | 68.0 | 37.2 | 1.0 | TKA | 40,491 | 287 | 6 |
| Durand, 2013 | France | 2003-2006 | 70.0 | 42.9 | 1.0 | TJA | 3,908 | 77 | 6 |
| Everhart, 2013 | USA | 2010-2012 | NR | NR | 1.0 | TJA | 1,875 | 29 | 6 |
| Namba, 2013 | USA | 2001-2009 | 67.4 | 37.0 | 1.0 | TKA | 56,216 | 404 | 6 |
| Bozic. 2014 | USA | 1990-2011 | NR | 55.0 | 3.7 | THA | 587 | 88 | 7 |
| Gou, 2014 | China | 2008-2010 | 53.7 | 38.8 | 1.1 | TKA and THA | 739 | 7 | 7 |
| Ravi, 2014 | Canada | 2002-2009 | 68.0 | 38.4 | 2.0 | TKA | 71,793 | 637 | 7 |
| Rasouli, 2014 | USA | 2010-2012 | 63.0 | 44.1 | 1.0 | TJA | 6,111 | 80 | 7 |
| Sousa, 2014 | UK, Portugal, and Spain | 2010-2011 | 68.0 | 37.0 | 1.0 | TKA and THA | 2,497 | 43 | 5 |
| Tai, 2014 | Australia | 1997-2003 | 67.8 | 46.4 | 10.0 | THA | 1,420 | 9 | 5 |
| Maoz, 2015 | USA | 2009-2011 | 61.6 | 45.9 | 2.0 | THA | 3,672 | 47 | 6 |

NR, not reported; UK, United Kingdom; USA, United States of America

**Figure A.** Relative risks of periprosthetic joint infection comparing males to females

CI, confidence interval (bars); Adjustment: +, unadjusted; ++, multivariate adjustment; †, are number of participants or arthroplasties

**Figure B.** Relative risks of periprosthetic joint infection comparing smokers to non-smokers

CI, confidence interval (bars); Adjustment: +, unadjusted; ++, multivariate adjustment; †, are number of participants or arthroplasties

**Figure C.** Relative risks of periprosthetic joint infection per 1 year increase in age

CI, confidence interval (bars); Adjustment: +, unadjusted; ++, multivariate adjustment; †, are number of participants or arthroplasties

**Figure D**. Relative risks of periprosthetic joint infection by body mass index comparisons

CI, confidence interval (bars); Adjustment: +, unadjusted; ++, multivariate adjustment; †, are number of participants or arthroplasties

**Figure E.** Relative risks of superficial wound infection by body mass index comparisons

CI, confidence interval (bars); Adjustment: +, unadjusted; ++, multivariate adjustment; †, are number of participants or arthroplasties

**Figure F.** Relative risks of periprosthetic joint infection comparing patients with diabetes versus no diabetes

CI, confidence interval (bars); Adjustment: +, unadjusted; ++, multivariate adjustment; †, are number of participants or arthroplasties

**Figure G.** Relative risks of periprosthetic joint infection comparing patients with rheumatoid arthritis versus no rheumatoid arthritis

CI, confidence interval (bars); Adjustment: +, unadjusted; ++, multivariate adjustment; †, are number of participants or arthroplasties

**Figure H.** Relative risks of periprosthetic joint infection comparing patients with history of steroid administration versus no history of steroid administration

CI, confidence interval (bars); Adjustment: +, unadjusted; ++, multivariate adjustment; †, are number of participants or arthroplasties

**Figure I.** Relative risks of periprosthetic joint infection comparing patients with osteoarthritis versus no osteoarthritis

CI, confidence interval (bars); Adjustment: +, unadjusted; ++, multivariate adjustment; †, are number of participants or arthroplasties

**Figure J.** Relative risks of periprosthetic joint infection comparing patients with cardiovascular disease versus no cardiovascular disease

CI, confidence interval (bars); Adjustment: +, unadjusted; ++, multivariate adjustment; †, are number of participants or arthroplasties

**Figure K.** Relative risks of periprosthetic joint infection for other medical and surgical history characteristics

CI, confidence interval (bars); Adjustment: +, unadjusted; ++, multivariate adjustment; IASI, intra-articular steroid injection; †, are number of participants or arthroplasties

**Figure L.** Risk of periprosthetic joint infection patients with diabetes versus no diabetes, grouped according to several study characteristics

CI, confidence interval (bars); Adjustment: +, unadjusted; ++, multivariate adjustment; IASI, intra-articular steroid injection; PJI, periprosthetic joint infection; THA, total hip arthroplasty; TKA, total knee arthroplasty; *, *P*-value for meta-regression; †, are number of participants or arthroplasties

**Figure M.** Assessment of small study effects by funnel plots and Egger’s regression symmetry tests

The dotted lines show 95% confidence intervals around the overall summary estimate calculated using a fixed effect model;; *P*-values for bias calculated using Egger’s test were 0.229; 0.657; 0.160; 0.120; 0.268; 0.441; < 0.001; 0.608; 0.325; 0.832; 0.199; and 0.121 for gender and PJI; smoking status and PJI; per 1 year increase in age and PJI; BMI ≥ 30 versus < 30 and PJI; BMI ≥ 40 versus < 40 and PJI respectively; BMI ≥ 30 versus < 30 and superficial wound infection; diabetes status and PJI; rheumatoid arthritis and PJI; steroid use and PJI; osteoarthritis and PJI; BMI, body mass index; CVD and PJI; and revision arthroplasty and PJI respectively; CVD, cardiovascular disease; PJI, periprosthetic joint infection
